# The Sufferings of Christ

**Published:** 1888-01-28

**Authors:** 


### Words of Consolation.

[Communications for this column should be addressed "Chaplain," care of the Editor of The Hospital, who will gratefully welcome any suggestions or inquiries from matrons, nurses, and the staff generally.]

### LXIV.—The Sufferings of Christ.

*For Reading to the Sick.*

"I thirst." (John xix. 28). No doubt there was something more than a feeling of bodily thirst expressed in these words. Jesus thirsted to do the whole will of his Father, for He said: "My meat is to do the will of Him that sent me, and to finish His work." (John iv. 34.) This was His spiritual thirst as well. He thirsted for the salvation of men, and for the full accomplishment of the scriptures which had spoken of His sufferings. No wonder then that He cried "I thirst" previous to the satisfaction proclaimed in the next cry from the Cross "It is finished." But we are still concerned with the bodily sufferings. We cannot but remember here one terrible sin of the flesh, the ruin of so many millions, England's special curse, the sin which brings sorrow, desolation, and disease to so many of our homes; that is, drunkenness. And should we not expect to find our Lord offering some very marked satisfaction for this? "My strength is dried up like a potsherd, and my tongue cleaveth to my gums, and Thou shalt bring me into the dust of death." (Psalm xxii. 15). What does all this agonizing drought upon the Cross correspond to but Nature's own, which is God's own, retribution for drunkenness? Is not this the physical penalty more or less attached to every degree of intemperance? We listen to that cry of loving mercy from the parched lips of the suffering Jesus, and as we listen we fancy we hear its echoes full of hope for the penitent drunkard. "See (He seems to say) I have borne thy curse. The pains which thou didst often try to alleviate by plunging yet deeper into sin, have been endured by one who came to deliver thee. I declined to taste even of vinegar in my innocence, to shew thee that the way of repentance can only be trodden by cutting thyself off from the cause of thy sin. Therefore when thou art in the hour of temptation refuse as I did." Otherwise, if there be no repentance after such a sacrifice, we look beyond the grave as we have in the case of other sins. There we hear another cry of agony, the cry of Dives. Dives may not have been a drunkard, but he was a sensualist. He was in the habit of faring sumptuously every day; and that always means indulgence of the flesh. Behold the forecast of hell. "Send Lazarus that he may dip the tip of his finger in water and cool my tongue, for I am tormented in this flame." (Luke xvi. 24).

Many are the efforts now being made to stem the fearful tide of intemperance. Temperance societies are making some headway against its ravages. Medical men are much more careful than they were about prescribing alcohol. The consumption of alcohol in our hospitals is yearly decreasing. Thousands of people who never were intemperate are more careful and drink much less than they did. May we venture to express a hope that as all, or any, of these efforts are extended, the great *motive* for repenting of drunkenness, or for becoming more temperate, may not be lost sight of, or the cause will degenerate into a question of simple utilitarianism. The motive for penitence in this case, as in all others, must be derived from the Cross. Let us take care that all those whom we would influence aright on the temperance question are *possessed* with that motive for their own sakes, and for others.

